# Deciphering the Neurotoxic Effects of *Karenia selliformis*

**DOI:** 10.3390/toxins17020092

**Published:** 2025-02-15

**Authors:** Ambbar Aballay-González, Jessica Panes-Fernández, Catharina Alves-de-Souza, Bernd Krock, Juan José Gallardo-Rodríguez, Nicole Espinoza-Rubilar, Jorge Fuentealba, Allisson Astuya-Villalón

**Affiliations:** 1 Laboratorio de Biotoxinas, Departamento de Oceanografía, Facultad de Ciencias Naturales y Oceanográficas, Universidad de Concepción, Concepción 4070409, Chile; amaballay@udec.cl (A.A.-G.); cathalves@udec.cl (C.A.-d.-S.); 2 Centro de Investigación Oceanográfica COPAS COASTAL, Universidad de Concepción, Concepción 4070409, Chile; bernd.krock@awi.de; 3 Laboratorio de Screening de Compuestos Neuroactivos, Departamento de Fisiología, Facultad de Ciencias Biológicas, Universidad de Concepción, Concepción 4070409, Chile; jpanes@udec.cl (J.P.-F.); nicespinoza@udec.cl (N.E.-R.); 4 Alfred-Wegener-Institut Helmholtz-Zentrum für Polar-und Meeresforschung, Am Handelshafen 12, D-27570 Bremerhaven, Germany; 5 Department of Chemical Engineering, Research Centre CIAIMBITAL, University of Almería, 04120 Almería, Spain; jgr285@ual.es; 6 Centro de Investigaciones Avanzadas en Biomedicina (CIAB-UdeC), Facultad de Ciencias Biológicas, Universidad de Concepción, Concepción 4070409, Chile

**Keywords:** dinoflagellate, harmful algal blooms, neurotoxic compounds

## Abstract

*Karenia selliformis* is a globally recognized dinoflagellate associated with harmful algal blooms and massive fish kills along southern Chilean coasts. Its toxicity varies with environmental factors and genetic diversity. While *K. selliformis* is traditionally linked to neurotoxins like gymnodimines (GYMs), analysis of the strain CREAN-KS02 from Chile’s Aysén Region (43° S) revealed no presence of toxins associated with this genus, such as gymnodimines, brevetoxins, or brevenal. Given the high toxicity and impact on marine life, our study aimed to functionally characterize the neurotoxic metabolites in the exudate of *K. selliformis* cultures. Cytotoxicity was evaluated using a Neuro-2a cell-based assay (CBA), determining an IC_50_ of 2.41 ± 0.02 μg mL^−1^. The incubation of Neuro-2a cells with the bioactive lipophilic extract obtained from the exudate of *K. selliformis* and the ouabain/veratridine couple showed activation of voltage-gated ion channels. Electrophysiological recordings on cultured mouse hippocampal neurons showed that the extract increased cell excitability in a dose-dependent manner, modulating action potential firing and exhibiting an opposed effect to tetrodotoxin. These findings indicate the presence of excitatory neurotoxic compounds affecting mammalian cells. This study provides the first mechanistic evidence of *K. selliformis* toxicity and highlights potential risks associated with its proliferation in marine environments.

## 1. Introduction

Fish-killing microalgal blooms have emerged as widespread global threats. These events have greater socio-economic impacts than the more extensively studied harmful algal blooms, which may cause seafood biotoxin contamination. *Karenia selliformis* is a dinoflagellate known worldwide for its association with ichthyotoxic harmful algal blooms. It has been responsible for massive fishkill events in southern Chile [1]. Regarding ichthyotoxins (i.e., bioactive compounds produced by microalgae that kill fish), the most common mechanism of killing fish is by damaging the sensitive gill membranes of fish (e.g., by karlotoxin, karmitoxin, prymnesins) [2]. However, additional evidence demonstrated that both direct and indirect exposure to *K. selliformis* induces pathological damage to the intestine and liver tissues of medaka fish, with varying degrees of severity [3].

It is well known that several species of *Karenia* produce neurotoxins. This species was primarily associated with the production of neurotoxins known as gymnodimines (GYMs) [4,5], characterized for targeting muscular and neuronal nicotinic acetylcholine receptors [6,7,8]. The analytical characterization of Chilean *K. selliformis* strains revealed the absence of gymnodimines as well as compounds such as brevetoxins and brevenal reported in other *Karenia* species [1]. Nevertheless, laboratory bioassays have shown the extreme toxicity of a Chilean *K. selliformis* strain on rainbow trout gill (RTgill-W1) cell line [9] as well as its allelopathic effect on other microalgae [10], suggesting the presence of uncharacterized toxins.

Despite the relevance of *K. selliformis* to aquaculture activities in Chile, due to the high mortality rates associated with their blooms in Patagonian fjords, little is still known about the toxic metabolites produced by this species and their cellular mechanisms of toxicity. Reports suggest that toxins produced by *K. selliformis* can be transferred through the food chain [3,11]. Therefore, it is crucial to elucidate the cellular effects of the toxins present in the crude exudates of Chilean *K. selliformis* strains to better understand the negative effects on aquaculture.

The Chilean *K. selliformis* CREAN_KS02 strain did not produce any known variants of gymnodimines or brevetoxins [1]. However, its extracts contained two compounds with the same mass transition as brevenal, a compound related to brevetoxins. Two peaks in the ion trace corresponding to brevenal’s mass transition (*m/z* 657.4 > 639.4) appeared at 3.39 and 3.61 min. This observation led to the collection of collision-induced dissociation (CID) spectra for these compounds. The CID spectra clearly demonstrated that neither compound matched brevenal, as their fragmentation patterns differed distinctly from brevenal’s spectrum (Figure 5 of [1]). These findings suggest the presence of previously unknown toxins. As many marine neurotoxins have been described to bind ion channels, specifically voltage-gated sodium channels (VGSCs) [12,13], we expect to observe modulation of receptors or channels due to the presence of potential neurotoxic compounds. If confirmed, this will represent a crucial step toward understanding their cytotoxic and neurotoxic effects and achieving their mechanistic characterization. This study aims to enhance the knowledge of the cellular and molecular effects of bioactive compounds present in the exudate of the *K. selliformis* strain CREAN-KS02, isolated from the Aysén Region, and to functionally characterize its neurotoxic metabolites.

## 2. Results

### 2.1. Effects of ExKs on Neuro-2a Cell-Based Assay (CBA)

**Figure 1:**
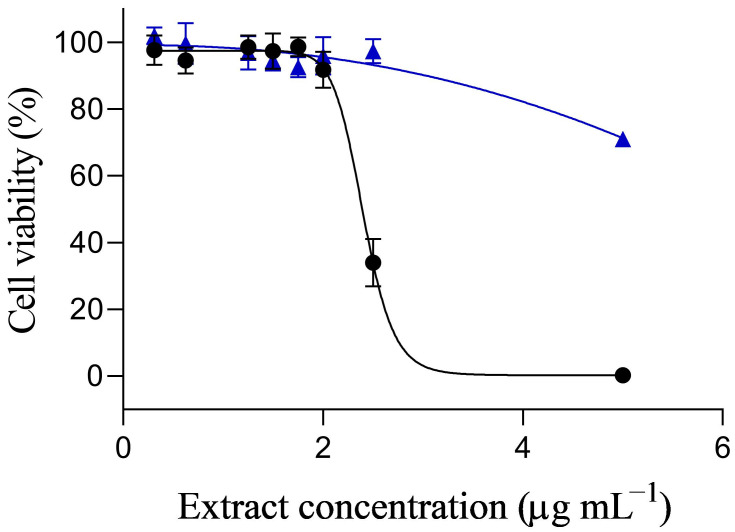
Cytotoxic curve of ExKs of Karenia selliformis (CREAN_KS02) on mouse neuroblastoma cells (Neuro-2a). The cells were incubated with extract of ExKs (0–5 µg mL^−1^) during 24 h (black dots, R^2^ = 0.985) or extract of culture medium (blue triangles). The calculated IC_50_ was 2.41 ± 0.02 µg mL^−1^. The graph shows three experiments performed in different weeks, with three replicates each one (*n* = 3, *N* = 3).

Using Neuro-2a cells, we tested the toxicity of bioactive lipophilic extract obtained from the exudate of *K. selliformis* cultures (ExKs) in a wide range of concentrations, observing that concentrations equal to or below 2 µg mL^−1^ of ExKs in culture medium, did not affect cell viability after treatments of 24 h (Figure 1). However, concentrations higher than 2 µg mL^−1^ showed a clear effect on viability with a drastic reduction in viability. At 2.5 µg mL^−1^ and 5 µg mL^−1^, viability values were 30 and 0.3%, respectively (Figure 1). The IC_50_ of ExKs, calculated after 24 h of exposure, was 2.41 ± 0.02 µg mL^−1^. The control extract, obtained from the culture medium without *Karenia* cells, exhibits cell viability above 90% across most of the concentration range used in this study (Figure 1). However, at 5 µg mL^−1^, it reduced viability to 70%. This may be associated with the effect of lipophilic compounds dissolved in seawater from the culture medium.

**Figure 2:**
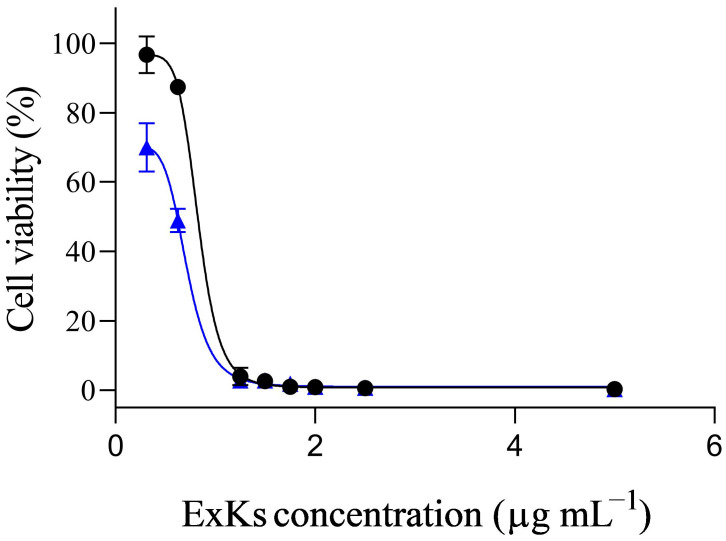
ExKs increases cytotoxicity in Neuro-2a cells in presence of ouabain (150 µM) and veratridine (15 µM). Neuro-2a cell assay for voltage-dependent sodium-channel-activating toxins exposed to ExKs, in the presence (black dot) or absence (blue triangles) of 100 nM saxitoxin. The graph shows a representative experiment of two experiments performed in different weeks, with three replicates each. (*n* = 2, *N* = 3).

The next step was to evaluate the effect of different ExKs concentrations on the cell viability of Neuro-2a cells incubated with ExKs in the presence of ouabain and veratridine. For that, Neuro-2a cells were treated with the Na^+^/K^+^-ATPase inhibitor ouabain (300 µM) and the sodium channel activator veratridine (30 µM M). The reduction in cytotoxicity in the presence of ouabain/veratridine or ‘cell rescue’ is indicative of a sodium-channel-blocking toxin (paralyzing toxins). Additionally, the enhancement of cytotoxicity in the presence of ouabain (150 µM) and veratridine (15 µM) is indicative of a potent sodium channel activator (brevetoxin-like toxins) [14,15]. An experiment was also carried out using saxitoxin (100 nM), incubated with the ExKs and ouabain and veratridine (150 µM/15 µM). The incubation of Neuro-2a cells with ExKs in the presence of 300 µM ouabain and 30 µM veratridine did not exhibit an effect (cell rescue) associated with blocking toxins (Supplementary Material S1). The presence of 150 µM ouabain and 15 µM veratridine potentiated over three times the cytotoxic effect of the ExKs extract (Figure 2, blue dots) with an IC_50_ at 0.71 µg mL^−1^. The black dot in Figure 1 shows that, especially at ExKs concentrations in the range of 0.3 to 2 µg mL^−1^, the cell viability was above 90% when ExKs was in the absence of ouabain and veratridine. In the presence of ouabain (150 µM) and veratridine (15 µM), proportional cell viability reduction was shown with increasing ExKs concentrations, reaching 2.8% at 1.25 µg mL^−1^ ExKs. These potentiation effects were dose-dependent, until 1.25 µg mL^−1^. Concentrations over this value decreased cell viability at all conditions. Upon co-incubation with 100 nM saxitoxin (Figure 2, black dots) and ExKs concentrations, the IC_50_ was 0.83 µg mL^−1^. At concentrations equal to or below 0.6 µg mL^−1^, the cytotoxic effect was reversed. However, at ExKs concentrations between 1.25 µg mL^−1^ and 2 µg mL^−1^, where ouabain- and veratridine-potentiated cytotoxicity exceeded 90%, no reversion of the cytotoxic effect by saxitoxin was observed.

### 2.2. Effect of ExKs on Synaptic Function and Excitability

**Figure 3:**
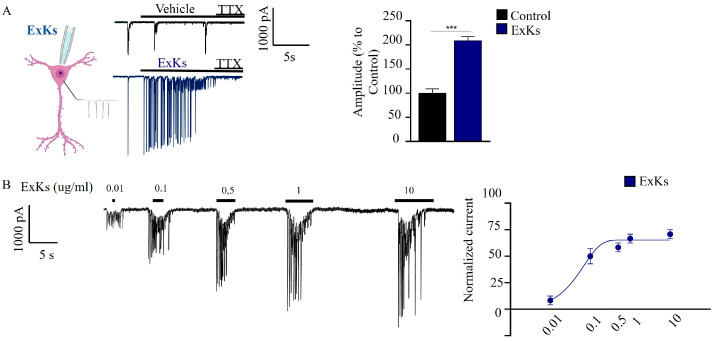
Karenia selliformis (CREAN_KS02) exudate extract (ExKs) induced a potentiation in the synaptic neurotransmission. (**A**) Illustrative scheme of perfusion for electrophysiological recordings. Representative traces of postsynaptic currents illustrate the effect of ExKs (1 µg mL^−1^) and TTX (50 nM) applications. The graph bars show the changes in the amplitude (pA) of the recordings (left panel). The values are mean ± SEM obtained from *n* = 8. (**B**) ExKs concentration–response curve, *n* = 7. The curve describes the potentiation of the neuronal synaptic currents elicited by ExKs (right panel) (*n* = 3, *N* = 12, *** *p* < 0.001).

To further investigate the effects of ExKs on neuronal synaptic function, a long-term culture of mice hippocampal cells (14 DIV) was electrophysiologically characterized under acute ExKs (1 µg mL^−1^) applications, and additionally, we evaluated the treatments using a perfusion chamber with sequential applications of different concentrations of extract (0.01–10 µg mL^−1^). Throughout the culture period, neurons showed a remarkable ability to reestablish neurites and synaptic connections, developing advanced electrophysiological properties. We applied ExKs to hippocampus neurons in an acute treatment. A TTX positive control (50 mM), serving as a voltage-gated sodium channel inhibitor, was also included. Spontaneous post-synaptic currents (sPSCs) using hole cells in the voltage-clamp configuration were used to register the currents under control conditions and acute exposition to ExKs (Figure 3A). Neurons treated with ExKs demonstrated a significative increase in global synaptic activity, and interestingly, the addition of TTX (µM) induced silencing of the synaptic activity observed in control conditions or after ExKs application. The amplitude of synaptic events in ExKs-treated neurons increased the amplitude of basal currents two-fold (C: 100 ± 9%; Ks 208 ± 8%).

**Figure 4:**
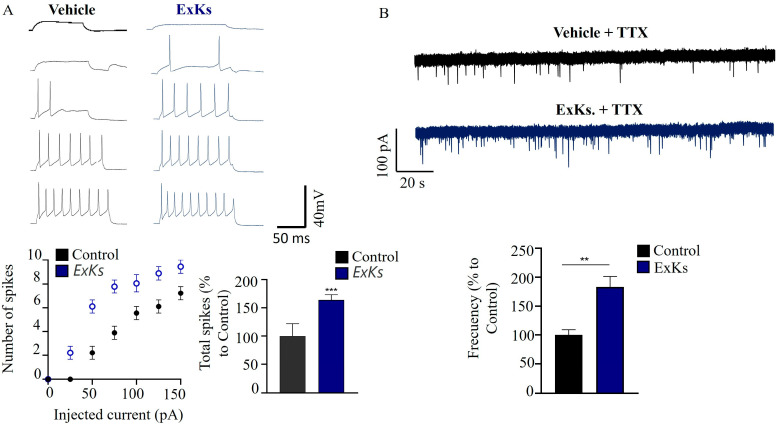
Modulation of excitatory neurotransmission by Karenia selliformis (CREAN_KS02) exudate extract (ExKs). (**A**) Current traces showing the comparison of APs firing in control and ExKs (1 μg mL^−1^) conditions, obtained in primary hippocampal neurons using electrophysiology patch clamp technique in current-clamp mode, and quantification of the relationship between the number of AP spikes and the injected current intensity, and the quantification of total spikes (bottom panel). The values represent mean ± SEM, obtained from *n* = 7 hippocampal neurons. Unpaired Student’s *t*-test was used for statistical analyses (*n* = 7–8 per group). (**B**) Miniature excitatory postsynaptic currents (mEPSC) were isolated using bicuculline (5 μM), and TTX (0.3 μM), and glutamatergic synaptic activity before and during the application of ExKs (1 μg mL^−1^). The bar plot summarizes the effects of ExKs on the amplitude of the frequency of mEPSC (Hz) in hippocampal neurons in vitro (bottom panel, *n* = 8). TTX (50 nM) was used as positive control (*n* = 3, *N* = 8,** *p* < 0.01, *** *p* < 0.001).

Regarding this significative effect on synaptic currents, we evaluated neuronal excitability, the capacity to fire the action potential (APs) and miniature postsynaptic excitatory activities (mEPSC). Analysis of action potentials (APs) using current clamp mode showed that the ExKs perfusion induced a persistent increase in the number of neurons with larger AP-amplitudes after progressive pulse amplitude, subsequently blocked by tetrodotoxin (TTX) pulse (Figure 4A). Notably, APs firing and the percentage of active cells appear to increase after acute ExKs perfusion, reflecting a potentially more sensitive condition of the neurons to develop APs and suggesting the possibility of neurotoxicity in the presence of ExKs. The comparison of properties for individual APs recorded suggests that the excitability was higher for ExKs-treated neurons for randomly sampled neurons (Figure 4A, Control: 100 ± 15%; ExKs: 163 ± 9%). This comprehensive analysis provides insight into this excitatory property of the ExKs to induce, in this way, damage to central synapses, providing the first evidence and valuable information for potential mechanisms.

In light of synaptic activity dynamics (specifically modulations in excitatory tone induced by ExKs), we meticulously employed TTX (50 nM) and bicuculline (5 μM) to selectively isolate miniature excitatory postsynaptic currents (mEPSC) in vitro, as illustrated in Figure 4B. The recorded traces demonstrate a significant increase in the frequency of excitatory synaptic currents subsequent to ExKs treatments (Control: 100 ± 9%; ExKs: 183 ± 18%).

### 2.3. ExKs Regulates Neurotransmitter Release via Ca^2+^ Handling

**Figure 5:**
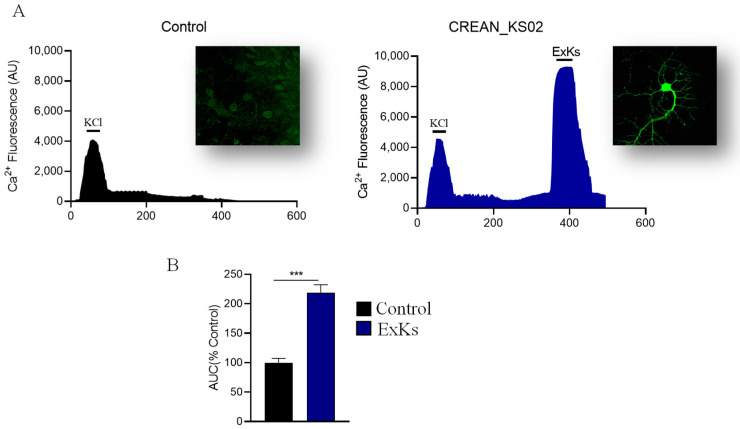
Karenia selliformis (CREAN_KS02) exudate extract (ExKs) induced Ca^2+^ influxes. (**A**) Representative traces of ExKs-induced (1 μg mL^−1^) changes in intracellular Ca^2+^ in hippocampal neurons after depolarizing stimulus of High K+ solution pulse (90 mM). (**B**) Quantification of areas under the curve (AUC) for each condition, *n* = 5–6. *** *p* < 0.001 compared to the control group.

Considering these findings, we sought to assess the connection between neurotoxicity and alterations in cytosolic Ca^2+^ levels in hippocampus neurons (Figure 5). To do this, FLUO-4TM-loaded neurons were acutely exposed to ExKs in the same previous conditions (Figure 5A). A pulse of high K^+^ solution (KCl, 90 mM) was used as depolarizing control, to check the standard neuron response; after that, the Ca^2+^ response to the application of ExKs was evaluated. The exposed neurons showed a strong, rapid and progressive increase in Ca^2+^ levels, significative higher than that observed with the KCl control (Figure 5B). This finding corroborates the potent effect of ExKs on neuronal excitability and cell activation, and eliciting Ca^2^⁺ increased those observed under control conditions over two-fold (depolarizing pulse).

**Figure 6:**
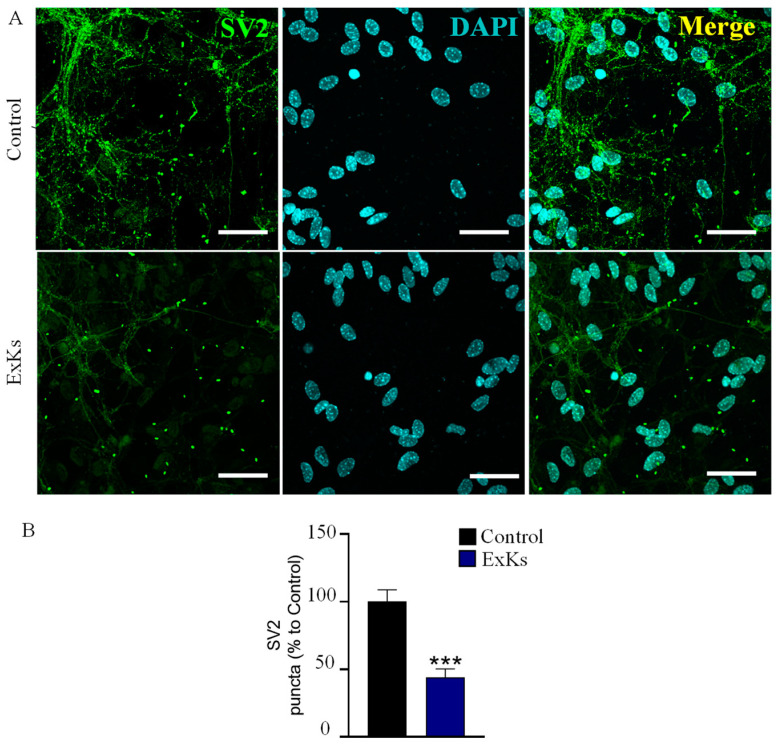
Karenia selliformis (CREAN_KS02) exudate extract (ExKs) induced SV2 depletion. (**A**) Representative confocal microscopy images show SV2 (green) and DAPI (cyan) staining. Top: control condition; bottom: acute treatment with ExKs (1 μg mL⁻^1^). (**B**) Quantification of the number of SV2 puncta in primary processes per 20 μm from panel A. Number of primary processes quantified: Control (21 processes from 15 neurons); ExKs (24 processes from 17 neurons). *** *p* < 0.001 compared to the control group.

The heightened excitability observed in the presence of the ExKs suggests a potential impact on neuronal and cellular signaling pathways. The disruption of Ca^2+^ homeostasis further implies a disturbance in the delicate balance essential for presynapses, since the function of exocytotic machinery is Ca^2+^-dependent. To test this hypothesis, we employed confocal imaging to assess the effects of ExKs on SV2 (Synaptic Vesicle Glycoprotein 2), a key regulator of vesicle trafficking and neurotransmitter release (Figure 6A). Our observations revealed that ExKs led to a strong reduction in the immunoreactivity of SV2, reflexed by a significative decrease in the number of puncta in the primary processes of these neurons (Figure 6B, Control: 100 ± 9%; ExKs.: 44 ± 6%), suggesting that the excitotoxic effects could induce depletion of presynaptic terminals.

## 3. Discussion

In the present study, we performed a functional characterization of compounds that were extracted from the exudate of a culture of *Karenia selliformis* (ExKs) and conducted a pharmacological comparison with the effects observed with tetrodotoxin (TTX) and saxitoxin (STX) using in vitro neuronal models. Yet uncharacterized compounds of *K. selliformis* CREAN_KS02 demonstrated high cytotoxicity in vitro assays on the rainbow trout gill (RTgill-W1) cell line, suggesting a significant potential for fish mortality and other marine species [1,16]. Notably, our results suggest that the ExKs extract induces a series of cellular responses with significant alterations in neurophysiological processes.

In our evaluation of the cytotoxic effects in the Neuro2A mammalian neuronal lineage model, we found that ExKs exhibits cytotoxicity in the murine model, with an IC_50_ of 2.41 ± 0.02 µg mL^−1^. Approximately the double concentration led to 100% lethality. This rapid neurotoxic effect is described as “on-off” or “fast-acting”, similar to spirolides and gymnodimines [17]. These results are consistent with those described by other authors regarding fish gill cell lines [1,9], and could help explain the massive deaths of several marine species associated with the participation of harmful algal bloom events (HABs) involving the species *K. selliformis* along the Chilean coasts [9].

Regarding its neurotoxic effect, we evaluate the presence of toxins capable of activating voltage-gated sodium channels (VGSCs) (similar to compounds like brevetoxin) or with sodium-channel-blocking activity (such as saxitoxin or tetrodotoxin) using the Neuro2a CBA model. As shown in Figure 2, Neuro-2a cells exposed to ExKs for 24 h exhibited a potentiating cytotoxic effect of veratridine and ouabain, consistent with the effect suggested for O/V-activating type toxins. Co-incubation with 100 nM saxitoxin resulted in a reduction in toxicity observed in the presence of ouabain/veratridine. These results indicate that VGSC blockage by STX interferes with the exacerbated toxicity of activating-type toxins previously observed with the ExKs extract. Since ExKs potentiates the toxicity of veratridine in the CBA model, which is expected for sodium-channel-activating toxins like brevetoxins, this implies that ExKs could be an agonist of the effect induced by ouabain/veratridine. Our results showed that neurons treated with ExKs demonstrated a significant increase in global synaptic activity, which was silenced by the addition of TTX (Figure 3A). Additionally, the increase in the amplitude of synaptic events and the persistent increase in the number of neurons with larger AP-amplitudes, which were subsequently blocked by a tetrodotoxin (TTX) pulse in ExKs-treated neurons (Figure 4A), suggest a positive modulation of neuronal activity. This heightened excitability may have potential implications for synaptic failures and the nuanced regulation of critical neural network functionality.

The alteration in cytosolic Ca^2^⁺ levels in hippocampal neurons when exposed to ExKs (Figure 5) has been observed in rat cerebellar granule neuron (CGN) cultures [18] and mouse cerebrocortical neurons [19] in response to exposure to VGSC-activating toxins such as PbTx-2. Calcium channels are crucial for synaptic vesicle exocytosis, and any interference at this level can lead to synaptic dysfunction, supporting the idea that ExKs may contribute to excitotoxicity and neural damage by potentiating calcium dyshomeostasis and altering neurotransmitter release, as seen in the significant reduction in SV2 immunoreactivity (Figure 6B). The observed potentiation in excitatory synaptic transmission suggests that the exudate extract enhances the acute efficiency of synaptic signaling, inducing a massive synaptic depletion (Figure 6), which could result in strong excitotoxicity and subsequent synaptic failure. This phenomenon draws intriguing parallels with other neurotoxic contexts, where enhanced neurotransmission may act as a precursor to neurodegeneration [20].

## 4. Conclusions

The data presented here shed light on several critical aspects of how *K. selliformis* exudate affects neuronal function. This study provides the first compelling evidence that exudates of this species exhibit significant neurotoxic effects mediated by potentiation of excitatory neural tone. The cell mortality was shown to increase in the presence of ExKs, from low concentrations and eliciting the maximal toxicity since the 1.25 µg/mL. Electrophysiological measures further confirm that these extracts disrupt the activity of electrically active mammalian cell membranes, exacerbating the impact of known excitatory toxins.

These results suggest the probable presence of novel neurotoxic compounds in *Karenia* exudates, warranting further investigation to identify and chemically characterize the compound, as well as to assess its impact on farmed fish.

## 5. Materials and Methods

### 5.1. Microalga Cultivation

The strain of the toxic dinoflagellate *Karenia selliformis* (CREAN-KS02), isolated from the Chilean Aysen Region at 43° S, was obtained from the Collection of Harmful Algae hosted at the Center for Harmful Algae Studies at the Chilean Institute for Fishery Development (CREAN/IFOP) and subsequently maintained in the Laboratory of Marine Biotoxins of the University of Concepción (LBTx-UdeC). The strain was grown in L1 medium prepared with seawater from the Chilean coast (36°31′ S, 73°08′ W), 0.2 µm filtered, and autoclaved, with the salinity previously adjusted to 32 PSU (salinity for which blooms of *K. selliformis* have been observed in the field [1]. Cultures were maintained at 17 °C under 140 μmol photon m^−2^ s^−1^ and 16 h/8 h light/dark photoperiod.

*K. selliformis* growth curves were determined from 1-L cultures initiated at 3000 cells mL^−1^, inoculated from stock cultures in the exponential phase. Cell density was evaluated daily using Sedgewick Rafter chambers [21]. The maximum growth rate (divisions per day) was estimated during the exponential growth phase (Stein, 1973) and was determined according to the following equation:

$$\mu^{-1}=\frac{1}{t_{2}-t_{1}}\times log\left(\frac{N_{2}}{N_{1}}\right)$$

where *µ*^−1^ is the growth rate, *N*_1_ is the initial cell concentration, *N*_2_ is the final cell concentration, *t*_1_ is the initial time, and *t*_2_ is the final time.

### 5.2. Extraction of Extracellular Bioactive Compounds from K. selliformis Exudates

Exudates of 1 L of *K. selliformis* culture in exponencial phase with a density of 20,000 cells mL^−1^ were collected by gentle vacuum filtration 400 mbar of cultures through glass fiber filters (0.7 µm nominal, grade F, Sterlitech) verifying under the microscope that the cells remain intact. In total, 10 g of adsorbent (diaion HP20 resin, Sigma-Aldrich, St. Louis, MI, USA) was used for extraction of the active compounds from the filtrate. Prior to application, the adsorbent was activated by gentle stirring in methanol overnight, followed by washing three times with distilled water. The activated adsorbent was added to the *K. selliformis* filtrate and was then incubated for 24 h with shaking (80 rpm) at room temperature. After incubation, the adsorbent was placed in a glass column with a fiberglass stopper. The filtrate was removed, and the column was washed with two column volumes of distilled water for the removal of salts. The elution was carried out with seven column volumes of methanol, the eluate that contained lipophilic compounds exuded by *Karenia selliformis* was collected, concentrated in a rotary evaporator at 40 °C, then dried at 40 °C in a nitrogen flow, weighed and dissolved in methanol.

This is referred to as bioactive lipophilic extract obtained from the exudate of *K. selliformis* cultures “ExKs”. As a negative control, the procedure described above was carried out with the culture medium.

### 5.3. Neuroblastoma Neuro-2a Cell Bioassay

The cell bioassay (CBA) was performed using the Neuroblastoma Neuro-2a cell following a protocol modified from [14]. The Neuro-2a cell line was obtained from the American Type Culture Collection (ATCC, CCL 131). Cells were maintained in RPMI-1640 medium supplemented with 1 mM sodium pyruvate, 2.5 μg mL^−1^ amphotericin B, 50 IU mL^−1^ penicillin, 50 μg mL^−1^ streptomycin, 2 mM glutamine and 5% fetal bovine serum, and stored at 37 °C in a humidified 5% CO_2_ atmosphere. Twenty-four hours prior to the assays, Neuro-2a cells were seeded in 96-well plates at 125,000 cells mL^−1^ in RPMI-1640 medium supplemented with (5%) fetal bovine serum and incubated under the same conditions described for cell maintenance.

For viability assays and IC_50_ determination, Neuro-2a cells were incubated for 24 h with eight concentrations of extracts ranging from 0, 0.3, 0.6, 1.25, 1.5, 1.75, 2.0, 2.5 and 5 µg mL^−1^. The methanol solvent content in each solution was less than 0.1% to avoid affecting the viability and functionality of the neuronal lineage.

A Neuro-2a cytotoxicity bioassay was performed to detect the presence of neurotoxins acting on VGSC. For the Neuro-2a CBA assay, cells were exposed to the extract along with ouabain/veratridine (150 µM/15 µM) to assess sodium-channel-activating toxins, or ouabain/veratridineO/V (300 µM/30 µM) to evaluate blocking toxins, in a final volume of 130 μL.

For both assays control Neuro-2a cells without extract were used. Additionally, to evaluate the competition of the ExKs with VGSC-blocking toxins, Neuro-2a cells were exposed to 150 µM ouabain, 15 µM, a range of concentrations from 0 to 5 µg mL^−1^ of ExKs and 100 nM of saxitoxin (National Research Council Cánada, Ottawa, Ontario, Canadá, CRM-STX-f).

After exposure, cell viability was assessed by using MTT (3-(4,5-dimethylthiazol-2yl)-2,5-diphenyltetrazolium bromide; Life Technologies, Carlsbad, CA, USA). The cells were incubated with 60 μL of RPMI-1640 medium (5% FBS) containing 0.83 mg mL^−1^ MTT for 30 min at 37 °C. Formazan salts were dissolved with 100 μL of dimethylsulfoxide, and absorbance was measured through spectrophotometry at 570 nm using a Synergy H1 Microplate Reader (Biotek, Winooski, VT, USA).

### 5.4. Primary Hippocampal Cultures

Primary cultures of embryonic hippocampi (E18) were obtained from pregnant C57BL/J6 mice treated in accordance with the regulations recommended by NIH (National Institute of Health, Bethesda, MD, USA) and the ethics committee at the University of Concepción. Mice were deeply anesthetized by CO_2_ inhalation before being sacrificed by cervical dislocation. Primary cultures of embryonic hippocampal were prepared as previously published [22] and plated at 320,000 cells mL^−1^ on coverslips coated with poly-L-lysine (Trevigen, Gaithersburg, MD, USA). Cultures were maintained at 37 °C with 5% CO_2_ for all experiments.

### 5.5. Electrophysiology

The whole-cell patch clamp technique was used as previously reported [22,23], using an Axopatch 200B amplifier (Axon Instruments, Union City, CA, USA) in voltage-clamp mode of −60 mV. Miniature post-synaptic currents (mEPSC) were pharmacologically isolated using appropriate concentrations of TTX (50 nM) (Alomone Labs, Jerusalén, Israel) and bicuculline (5 μM). TTX was purchased from Alomone Labs (Jerusalem, Israel) and bicuculline was from Tocris (Bristol, UK). The pipette solution was (in mM) 120 KCl, 4 MgCl_2_, 10 HEPES, 2 ATP, 0.5 GTP, and 10 BAPTA (pH 7.4, 300 mOsm). The bath solution contained (in mM) 150 NaCl, 5.4 KCl, 2 CaCl_2_ 2,1 MgCl_2_, 10 HEPES and 10 Glucose (pH 7.4, 320 mOsm). Analysis was performed offline using Clampfit 10 (Axon Instruments, Union City, CA, USA).

To maintain a net electric current equal to zero for the “voltage clamp” mode, an external solution of fixed composition (in mM: NaCl 150; KCl 5.4; CaCl_2_ 2; MgCl_2_ 1; Glucose 10; HEPES 10; pH 7.4; osmolarity 300 mOsm) is used, while patch microelectrodes are filled with an internal solution of defined composition in mM: KCl 120; MgCl_2_ 4; HEPES 10; ATP 2; GTP 0.5; pH 7.4; osmolarity 300 mOsm. For the “current clamp” mode, the external and internal solutions were prepared with different ion concentrations to simulate normal physiological conditions. Patch microelectrodes were filled with an internal solution of defined composition in mM: 114 K-Gluconate, 4 KCl, 4 MgCl_2_, 10 BAPTA, and 10 HEPES (pH 7.4 adjusted with KOH, 290 mOsm L^−1^) for the “current clamp” mode, and they have an approximate resistance of 4–6 MΩ. To study the membrane potential (Vm), action potentials (APs) were elicited using a series of continuous current pulses applied for 300 ms (from −300 pA to +275 pA, increasing in steps of 25 pA).

### 5.6. Ca^2+^ Measurements

Hippocampal neurons plated in bottom transparent 96-well black plates and loaded with the non-ratiometric cytosolic and mitochondrial Ca^2+^ sensitive fluorescent probe Fluo4-AM 3 μM (Invitrogen, Carlsbad, CA, USA) for 30 min at 37 °C in PBS, using standard incubation conditions. Subsequently, the cells were washed for 20 min with PBS pH 7.4, washed 3 times with normal external solution, and finally mounted on a Nikon TE-200-U inverted microscope (Tokyo, Japan). Wavelengths of excitation and emission were 494/506 nm (Fluo-4 AM). ExKs-induced responses were expressed as a percentage of the fluorescence value at each time point (% Ft). This was calculated by subtracting the fluorescence at a given time (Ft) from the basal fluorescence value before (F0) and dividing by the F_max_ F_min_ fluorescence. The maximum % fluorescence intensity for Fluo-4 (maximum %[Ca^2+^]c) was obtained for each experiment.

### 5.7. Immunocytochemistry

The cells were washed with PBS 1X and fixed with 4% paraformaldehyde for 10 min at room temperature (RT). Later, cells were permeabilized with 0.1% Triton X-100 and blocked with 10% horse serum for 30 min at RT. Samples were incubated for 1 h at RT with the primary antibodies: anti-SV2 (1:200, Synaptic system, rabbit). Subsequently, the corresponding Cy3 or Alexa Fluor 488 conjugated antibodies were incubated for 45 min. Then, the samples were incubated with 300 nM DAPI nuclear staining for 10 min and mounted using DAKO immunofluorescence mounting media (Dako, Glostrup, Denmark). Images were acquired using an LSM780 NLO confocal microscope (Carl Zeiss Microscopy, Jena, Germany). The 63X/1.4 NA oil immersion objective with an additional 1.6X lens was used which explains why the total increase was 1008X. Images were processed, and quantification was performed using Image J (NIH Bethesda, Bethesda, MD, USA). To analyze SV2 puncta, we use the “Analyze Particles” tool from Image J tool, with size parameters set between 0.1 µm^2^ and 3 µm^2^ to capture relevant synaptic signals. Circularity was set between 0.3 to 1 to exclude artifacts. The total number of puncta per field was normalized to the area of the region of interest (ROI) as well as the number of cells manually counted in each field.

### 5.8. Data Analysis

The obtained data was analyzed using GraphPad Prism 6 Software. Normality was verified using the Kolmogorov–Smirnov test or Shapiro–Wilk normality test depending on sample size. Statistical significance was determined using unpaired Student’s *t*-test (for two groups) or one-way ANOVA followed by Dunnett’s multiple comparisons test (for multiple group comparisons). All experimental data were expressed as mean ± standard error of the mean (SEM) (unless otherwise indicated). *p*-values < 0.05 were considered statistically significant.

## Data Availability

The original contributions presented in this study are included in the article/supplementary material. Further inquiries can be directed to the corresponding author(s).

## Supplementary Materials

The following supporting information can be downloaded at: https://www.mdpi.com/article/10.3390/toxins17020092/s1. Figure S1: Neuro-2a cell bioassay controls.

## Abbreviations

The following abbreviations are used in this manuscript:

CBA	Cell-based assay
ExKs	The bioactive lipophilic extract derived from the exudate of *Karenia selliformis* strain (CREAN-KS02)
STX	Saxitoxin
TTX	Tetrodoxin
VGSC	voltage-gated sodium channels (VGSCs)
