# Supplementary material for: Deciphering the Neurotoxic Effects of Karenia selliformis
Source: Toxins (Basel). 2025 Feb 15;17(2):92. doi: 10.3390/toxins17020092 (PMC11861105; doi:10.3390/toxins17020092)
Supplement: Supplementary file 1 [file toxins-17-00092-s001.zip › toxins-3441310-supplementary.pdf]

Supplementary Material: Deciphering the Neurotoxic Effects of *Karenia selliformis*

Ambbar Aballay-González, Jessica Panes-Fernández, Catharina Alves-de-Souza, Bernd Krock, Juan José Gallardo-Rodríguez, Nicole Espinoza-Rubilar, Jorge Fuentealba and Allisson Astuya-Villalón

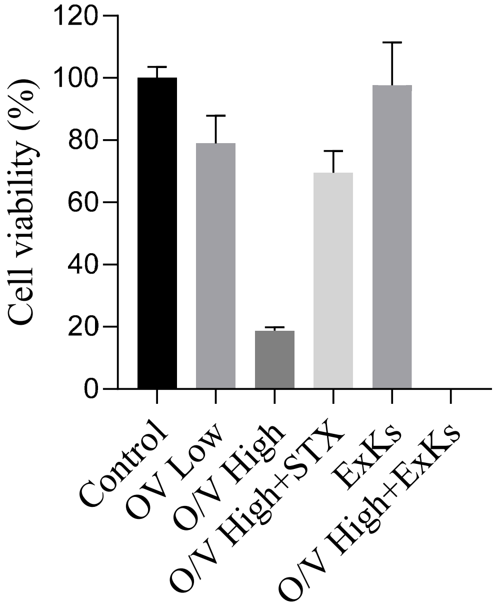

Control	Culture medium
O/V Low	150 uM Ouabain/ 15 uM Veratridine
O/V High	300 uM Ouabain/ 30 uM Veratridine
O/V High+STX	300 uM Ouabain/ 30 uM Veratridine + STX 100uM
ExKs	0.3 µg*mL^{-1}
O/V High+	300 uM Ouabain/ 30 uM Veratridine + 0.3 µg*mL^{-1}

Figure S1. Neuro-2a cell bioassay controls.
